# Factors Predicting In-School and Electronic Bullying among High School Students in the United States: An Analysis of the 2021 Youth Risk Behavior Surveillance System

**DOI:** 10.3390/children11070788

**Published:** 2024-06-28

**Authors:** Tran H. Nguyen, Gulzar H. Shah, Ravneet Kaur, Maham Muzamil, Osaremhen Ikhile, Elizabeth Ayangunna

**Affiliations:** 1 Department of Health Management, Economics & Policy, School of Public Health, Augusta University, Augusta, GA 30912, USA; 2 Department of Health Policy and Community Health, Jiann-Ping Hsu College of Public Health, Georgia Southern University, Statesboro, GA 30460, USA; 3 Department of Family and Community Medicine, College of Medicine, University of Illinois, Rockford, IL 61107, USA; 4 Department of Education, Kinnaird College for Women’s University, Lahore 54000, Pakistan

**Keywords:** bullying, in-school bully, cyberbullying, youth, risk factors

## Abstract

Background: Bullying is a global public health problem with severe adverse effects on behavioral health. Understanding the predictors of victimization by bullying is essential for public policy initiatives to respond to the problem effectively. In addition to traditional in-person bullying, electronic bullying has become more prevalent due to increasing social interaction and identity formation in virtual communities. This study aims to determine the predictors of in-school and electronic bullying. Methods: We employed multivariable logistic regression to analyze a nationally representative sample of 17,232 high school students in the United States, the 2021 Youth Risk Behavior Surveillance System national component. The survey was conducted during the COVID-19 pandemic, from September through December 2021. The factors examined included sociodemographic characteristics (age, gender, race), appearance (obesity), physically active lifestyles (being physically active, spending a long time on digital games), and risk-taking behavior (using marijuana). Results: Our results indicated that sociodemographic characteristics were strong predictors of being bullied in school and electronically. Being obese is more likely to result in bullying in school (AOR = 1.32, *p* = 0.003) and electronically (AOR = 1.30, *p* = 0.004). Adolescent students showing marijuana use had higher odds of being bullied in school (AOR = 2.15, *p* < 0.001) and electronically (AOR = 1.81, *p* < 0.001). While spending a long time on digital devices raises the risk of being electronically bullied (AOR = 1.25, *p* = 0.014), being physically active is not associated with being bullied. Neither of the two lifestyle factors was associated with in-school bullying. Conclusions: Interventions addressing violence among adolescents can benefit from empirical evidence of risk factors for bullying victimization in high school.

## 1. Introduction

Bullying is a global public health problem, with severe health consequences not only for victims but for all who are involved, perpetrators and bystanders [1,2,3]. There are three common types of bullying: physical, verbal, and social [3,4]. With the exponential growth in the use of social media for the social interaction and identity formation readily available in virtual communities, bullying through electronic media is becoming ever more prevalent. This is known as electronic bullying or cyberbullying, defined as repetitive aggressive behavior toward others using technology such as cell phones, computers, and other electronic devices [5]. Bullying is one of the significant risk factors for poor physical and emotional health, as it increases the risk for depression, anxiety, self-harm, sleep disturbances, and poor academic performance [5]. Given that the health effects of bullying are both short-term and appear later in life, it is important to understand bullying to respond effectively. 

Studies examining predictors for bullying have focused on specific demographic and physical characteristics. Researchers have found that demographic factors such as age, gender, ethnicity, and race are linked to bullying, although their findings are mixed. Some studies have indicated that boys are more likely to engage in physical and verbal bullying, while girls are more likely to experience cyberbullying [6,7,8]. Moreover, some studies have not found a statistically significant association between gender and cyberbullying [9,10].

The association between race and bullying experiences shows inconsistent patterns. According to the National Center for Education Statistics (NCES), an equal proportion of White and Black students (23%) reported being bullied at school, compared to a lower proportion of Hispanic students (16%) [11]. Other studies have shown different findings. Some studies found that Black students reported fewer experiences of bullying than White students [12,13]. Also, a survey of school climate perceptions found that minoritized racial and ethnic groups negatively influence the connection between school climate and experiences of bullying victimization [14].

Bullying is not confined to individuals with specific sociodemographic groups. Other individual-level factors have been linked to bullying. Obesity, a common reason for adolescents to be teased, is a big concern due to the increasing number of obese children in the United States (U.S.) and worldwide [15,16]. Researchers have found a significant connection between obesity and higher rates of bullying among children, especially boys [17,18]. These findings are consistent with a review of studies published between 2006 and 2016, which showed that obese and overweight children are at a higher risk of being bullied or victimized by their peers [19].

The widespread use of electronic devices like smartphones, tablets, gaming consoles, and computers has raised health concerns. In the U.S., children aged 8–12 and teens spend an average of 9 h on screens daily [20]. Research has shown that increased screen time is linked to various adverse health behaviors among young people, including poor sleep quality [21], lower academic performance [22], poor mental health [23], and reduced physical activity [24]. Studies on the relationship between screen time and bullying are limited, and mainly from outside the U.S. [25,26]. Prolonged screen time leads to a sedentary lifestyle, while physical activity is a protective factor against bullying [27]. Research indicates that not meeting the target of at least 1 h of moderate-to-vigorous physical activity per day, recommended by the World Health Organization (WHO), is associated with increased bullying victimization [28]. Furthermore, the prevalence of marijuana use among teens has risen significantly, with negative impacts on school performance, social life, and mental health [29,30]. Marijuana use has also been connected to cyberbullying victimization [31].

In addition, in March 2020, the WHO declared a severe acute respiratory syndrome coronavirus 2 (SARS-CoV-2) outbreak, a pandemic named COVID-19. The pandemic has had an unprecedented impact on the lives of school-age children worldwide, primarily due to prolonged school closures. Studies conducted globally have highlighted the significant burden of the pandemic on their education, quality of life, social interactions, and health, particularly their mental well-being [32,33]. Researchers worldwide have also examined the effects of COVID-19 on the prevalence of bullying. While the pandemic has reduced the prevalence of in-school bullying, the rate of cyberbullying does not seem to be affected [34,35,36,37].

This study aims to address several gaps in the literature related to bullying. First, there are inconsistent findings in studies exploring the relationship between bullying and sociodemographic factors such as gender and race/ethnicity. Second, the association of bullying with other unhealthy behaviors, such as lack of physical activity, marijuana use, and prolonged computer screen time, has been rarely explored in the U.S. Third, while marijuana use has been investigated as an outcome of bullying, it has not been studied as a predictor of bullying. Fourth, there is a lack of studies exploring bullying and its predictors based on recent datasets representing the U.S. adolescent population. Fifth, the association between the recent COVID-19 pandemic and youth bullying has not yet been studied. The current study examines the predictors for being bullied in school or electronically. We hypothesize that demographic characteristics (age, gender, and race), appearance (obesity), a physically active lifestyle (being physically active, spending a long time on digital games), and risk-taking behavior (e.g., using marijuana) are associated with high school students being bullied in school or electronically. In this paper, the terms cyberbullying and electronic bullying are used interchangeably.

## 2. Materials and Methods

### 2.1. Data Source

This study used a quantitative, cross-sectional study design based on secondary data from the 2021 Youth Risk Behavior Surveillance System (YRBSS), which is part of a series of biennial national school-based surveys conducted in both public and private schools by the Centers for Disease Control and Prevention (CDC). The survey monitors risky health behaviors that cause morbidity and mortality among middle and high school students in the U.S. The YRBSS comprises surveys from national, state, territorial, and tribal governments, as well as local schools. The 2021 survey used a three-stage cluster sampling design to select 209 schools from a sampling frame of all grades 9 to 12 students in public and private schools in the 50 states and the District of Columbia. Valid responses from 17,232 students were collected using self-administered questionnaires, with a school response rate of 72.7%, a student response rate of 79.1%, and an overall response rate of 57.5%. Additional information on the YRBSS is available elsewhere [38]. 

### 2.2. Variables

The study had two dependent variables—in-school bullying and cyberbullying. The in-school bullying variable was operationalized by asking if the student had ever been bullied on school property during the 12 months before the survey, with the response choices of no or yes. The cyberbullying variable was operationalized by asking students if they had ever been electronically bullied through texting, Instagram, Facebook, or other social media during the 12 months before the survey, with the response choices of no or yes. 

The independent variables included sociodemographic characteristics (*age, gender, race*), appearance (*obesity*), physically active lifestyles (*being physically active, spending a long time on digital games*), and risk-taking behavior (e.g., *using marijuana*). Table 1 details the descriptions of the dependent and independent variables. 

### 2.3. Analysis

A descriptive analysis was conducted to determine the frequencies and percentages of both dependent and independent variables. Chi-square tests were performed to examine the association of each independent variable with the dependent variables. Two models of multivariable logistic regression were run to determine the predictors of cyberbullying and in-school bullying. Both logistic models contain all independent variables indicated in Table 1. The significance threshold was set at 95%. All analyses were performed using STATA version 18 software, which is recommended by the CDC as one of the statistical software packages that account for the complex sampling design by YRBSS. 

## 3. Results

**Figure 1:**
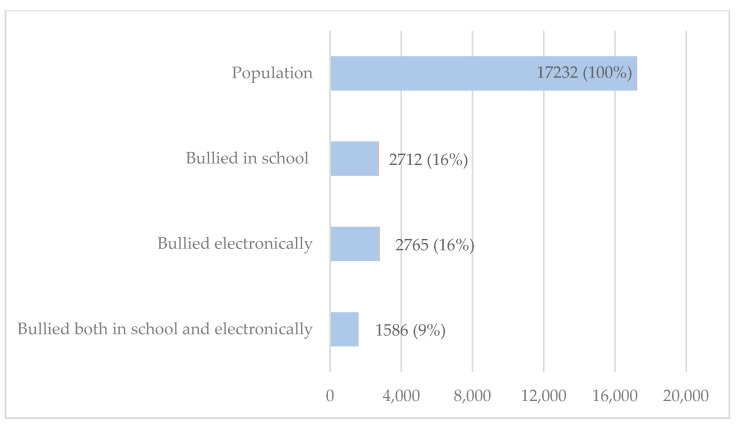
Percentage of U.S. high school students reporting being bullied, YRBSS, 2021.

Figure 1 illustrates the proportions of students who reported being cyberbullied or in-school bullied. About 16% of students reported being bullied in school, and 16% of students also reported being bullied electronically, with 9% of students reporting being bullied both in school and electronically.

Table 2 presents the descriptive characteristics of the variables. Almost equal proportions of students who responded to the survey reported being bullied in school or electronically. Of the 17,232 students who participated in the survey, the age groups less than 17 were distributed almost equally. Twenty percent (20.45%) of participants were 14 years old or younger, 25.84% were 15, 24.96% were 16, and 22.79% were 17. The 18 or older age group accounted for roughly 6% (5.97%). Forty-eight percent of the participants were female, while 51.96% were male. The majority of the participants were White (54.47%), followed by Multiracial (18.04%), Black (13.82%), and Hispanic/Latino (7.22%). About 17.15% of participants perceived themselves as obese. Over 54% reported being physically active for at least an hour a day for more than 5 days for the past 7 days. A staggering number of 75.36% of respondents reported spending more than 3 h a day on video/computer games or computers, while 15.67% reported currently using marijuana.

The Chi-square analysis found that all independent variables have statistically significant associations with being in-school bullied, while the majority of independent variables have statistically significant associations with being cyberbullied, except obesity (see Table 3).

Table 4 displays the logistic regression analysis predicting victimization through cyberbullying and in-school bullying. 

### 3.1. Cyberbullying Model

**Figure 2:**
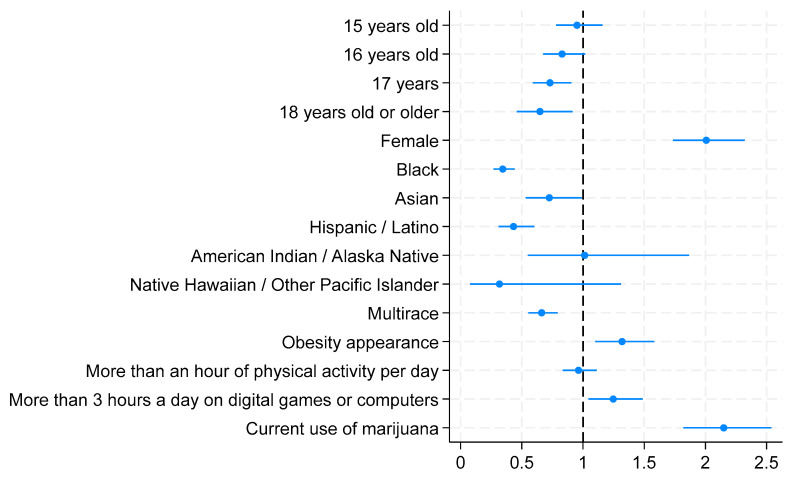
Adjusted odds ratios from logistic regression model predicting cyberbullying (Forest Plot).

Compared with the age group of 14 years and younger, the age groups of 15 and 16 years old did not show significant associations with being cyberbullied. In contrast, the age groups of 17 years and 18 years and older displayed statistically significant associations with cyberbullying. The older the students were, the less likely they were to be cyberbullied. Compared to the age group of 14 years and younger, the age group of 17 years was 27% less likely to be cyberbullied (AOR = 0.730; 95% CI = 0.491–0.749; *p* = 0.004), while the age group of 18 years and older was 55% less likely to be cyberbullied (AOR = 0.648; 95% CI = 0.459–0.914; *p* = 0.014). Females were found to be two times more likely to be cyberbullied compared to their counterparts (AOR = 2.001; 95% CI = 1.735–2.322; *p* < 0.001). In comparison to White, non-White racial groups were found to be less likely to be cyberbullied, except the American Indian or Alaska Native and Native Hawaiian or other Pacific Islander groups, who did not present significant associations. Blacks were 65% less likely than Whites to be cyberbullied (AOR = 0.344; 95% CI = 0.268–0.442; *p* < 0.001), while Asians were 28% less (AOR = 0.724; 95% CI = 0.530–0.990; *p* = 0.043), Hispanics/Latinos were 57% less (AOR = 0.432; 95% CI = 0.310–0.602; *p* < 0.001), and multiracial students were 34% less likely to be cyberbullied (AOR = 0.662; 95% CI = 0.553–0.793; *p* < 0.001). Obesity appearance was statistically associated with being cyberbullied. Students who appeared obese had 32% higher odds of being bullied at school than non-obese students (AOR = 1.319; 95% CI = 1.010–1.582; *p* = 0.003). While being physically activity for more than an hour per day for 5 days during the past 7 days did not show a statistically significant association with cyberbullying, playing video/computer games or using a computer for more than 3 h per day was statistically significantly associated with cyberbullying. Students who spent more than 3 h per day playing digital games or using computers had 25% higher odds of being bullied at school compared to those who did not (AOR = 1.247; 95% CI = 1.046–1.487; *p* = 0.014). Students who used marijuana were two times more likely to be cyberbullied than those who had never used marijuana (AOR = 2.150; 95% CI = 1.820–2.539; *p* < 0.001). Figure 2 presents a graphical display of the adjusted odds ratios from the logistic model predicting cyberbullying using a Forest Plot. 

### 3.2. In-School Bullying

**Figure 3:**
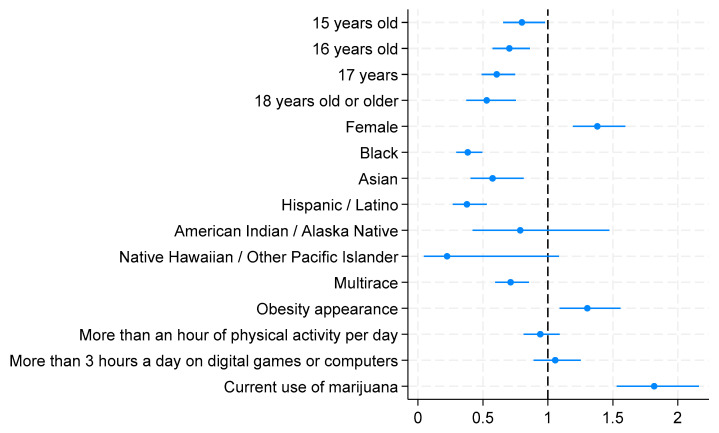
Adjusted odds ratios from logistic regression model predicting in-school bullying (Forest Plot).

Compared with the age group of 14 years and younger, the older age groups of 15, 16, 17 years, and 18 years and older showed statistically significant associations with in-school bullying. The older the students were, the less likely they were to be in-school bullied. Compared to the age group of 14 years and younger, the age group of 15 years was 20% less likely to be in-school bullied (AOR = 0.800; 95% CI = 0.655–0.977; *p* = 0.028), while the age group of 16 years was 30% (AOR = 0.703; 95% CI = 0.574–0.861; *p* = 0.001), that of 17 years was 40% (AOR = 0.606; 95% CI = 0.491–0.749; *p* < 0.001), and that of 18 years and older was 47% (AOR = 0.529; 95% CI = 0.372–0.753; *p* < 0.001) less likely to be in-school bullied. Females were found to be 1.38 times or 38% more likely to be in-school bullied compared to their counterparts (AOR = 1.380; 95% CI = 1.193–1.597; *p* < 0.001). Compared to the White group, the non-White racial groups were found to be less likely to be in-school bullied, except the American Indian or Alaska Native and Native Hawaiian or other Pacific Islander groups, who did not display significant associations. Blacks were 62% less likely than Whites to be in-school bullied (AOR = 0.383; 95% CI = 0.296–0.495; *p* < 0.001), while Asians were 43% less (AOR = 0.574; 95% CI = 0.405–0.814; *p* = 0.002), Hispanics/Latinos were 62% less (AOR = 0.376; 95% CI = 0.0.268–0.530; *p* < 0.001), and multiracial students were 29% less likely to be in-school bullied (AOR = 0.7132; 95% CI = 0.594–0.855; *p* < 0.001). Obesity appearance was statistically associated with being in-school bullied. Students who appeared obese had 30% higher odds of being bullied at school than non-obese students (AOR = 1.303; 95% CI = 1.090–1.559; *p* = 0.004). Neither physical activity for more than an hour per day for 5 days during the past 7 days nor playing video/computer games or using a computer for more than 3 h per day was statistically significantly associated with in-school bullying. Students who used marijuana were 18% more likely to be in-school bullied than those who had never used marijuana (AOR = 1.818; 95% CI = 1.529–2.162; *p* < 0.001). Figure 3 presents a graphical display of the adjusted odds ratios from a logistic model predicting in-school bullying using a Forest Plot. 

## 4. Discussion

Bullying can lead to serious negative physical and mental health outcomes for young people. This makes it a serious public health concern [1]. The adverse impact of this problem on students, families, and society merits the analysis of demographic and other behavioral risk factors of victimization through cyberbullying or in-school bullying. The current study showed that these demographic and behavioral risk factors were mostly similar for both in-school and electronic bullying. The odds of being bullied in school declined with age, which is consistent with a global pattern that reveals bullying peaks at about 12 years of age [39,40]. While in-school bullying tends to reduce with the increasing age of the victim, electronic bullying appears to be fairly consistent, irrespective of age group. Studies elsewhere show that young adults older than 18 years still experience electronic bullying more than the older age group of 66 years and older, and it should not be surprising that adolescents experience electronic bullying irrespective of age [41].

While the existing research has shown mixed results regarding the role of the victim’s gender in bullying, the current study provided evidence that females have significantly higher odds of being bullied in person and electronically. Some research studies suggest that although males are more likely to be the perpetrators of bullying, the likelihood of being a victim of in-person bullying reduces as they grow older [42]. This could be because male adolescents feel they can defend themselves as they grow older and instead may believe that showing aggressive behaviors becomes more acceptable [43,44]. Females also have an increased risk of being victims of electronic bullying because of their increased use of social media and time spent alone [45].

Our findings showed that White adolescents have higher odds of being in-school bullied and cyberbullied than Black, Asian, or Hispanic/Latino adolescents. This finding is consistent with existing studies that analyzed previous YRBSS surveys. Even though White students tend to be at a higher risk of being bullied, Black and Latino students were less likely to report in-school or electronic bullying [46]. This study found that obese students had higher odds of being in-school and electronically bullied, which is consistent with a multinational study in other countries [47,48,49]. Weight was found to be a major reason for bullying. Being obese can lead to stigmatization from peers, which makes obese adolescents more likely to be bullied [42,43]. In our study, being physically active was not a significant predictor of either in-school or electronic bullying, while another study found that being physically active was associated with lower risks of being a bully victim [49]. Spending long hours on the computer leads to a sedentary lifestyle, which is a risk factor for obesity. So, it is not surprising that we found that playing video and computer games and using a computer for more than three hours a day placed young people at risk of being bullied in school and electronically. Our finding is consistent with a study that found boys who played video games excessively were more likely to be bullied [18]. While we found that using marijuana was associated with both in-school and electronic bullying, an earlier study found that only electronic bullying was associated with marijuana use [50,51,52].

Notable was that the prevalence of in-school bullying decreased from 20% in 2019 to 16% in 2021 [45]. This improvement can be explained by the time the survey was conducted. The current YRBSS data was collected during the COVID-19 pandemic in 2021, when K-12 schools nationwide were not fully returning to in-person learning [53]. Also, the pandemic may impact the prevalence of physical activity and spending time on digital devices, which increased from 21.6% to 54.0% for being physically active more than an hour a day and from 43.4% to 75.4% for spending more than 3 h a day on digital devices [54]. While being physically active lowers the risk of being bullied, spending more time on digital devices raises the risk [55,56]. More time on the internet can raise the risk of victimization, particularly through cyberbullying, attributable to broader exposure, leading to more interactions and increased chances of encountering bullies who will gather more information to exploit their victims. Given the rapid rise in Artificial Intelligence (AI) applications and their implications for teaching and learning practices, leading to the increasing use of the internet and digital devices in education [57], future studies should focus on understanding the use of screen time for academic compared to non-academic purposes and the risk of bullying.

Our study findings have several latent implications for schools and other stakeholders, such as behavioral health organizations. For instance, it provides nationwide data-driven evidence for schools in the United States to create interventions tailored to students’ sociodemographic characteristics, taking into account age, gender, and race to personalize support and prevention strategies. Such interventions can benefit from our findings in creating inclusive policies that promote body positivity and race-related inclusivity, encouraging schools to strategize stigma reduction and encourage acceptance of racial diversity. Our study can also inform community-level anti-bullying initiatives by behavioral health organizations, to serve as resources for students who are the victims of bullying, including counseling services, digital literacy programs, and support groups customized for students most vulnerable to bullying. 

Our study has several limitations. Analyzing a secondary dataset means our study is subject to the disadvantages that come with this design. Additionally, the respondents are school students, so it does not capture young people who are out of school or homeschooled. Moreover, the YRBSS is a cross-sectional study, so our study cannot establish causality between the variables of interest and in-school or electronic bullying. Finally, the YRBSS survey is self-reported and has several limitations, so our study inherits these limitations. Regardless of the above-mentioned limitations, the current study’s findings significantly contribute to the existing literature on the topic. One major strength is that our research analyzed a nationally representative sample, increasing the results’ generalizability. By examining the 2021 YRBSS, which was conducted during the COVID-19 pandemic, our study highlights the impact of the pandemic on high school students’ risk behaviors and offers evidence to address the multifaceted public health needs of young people. 

## 5. Conclusions

In line with the study’s objectives, our study sheds light on the complex interplay between sociodemographic and lifestyle factors and the prevalence of in-school bullying and cyberbullying. These findings underscore the importance of addressing bullying as a multifaceted public health problem, taking into account the intersectionality of risk factors that contribute to its prevalence. The study’s insights into race, age, and gender dynamics offer valuable guidance for developing targeted interventions to protect vulnerable groups from the adverse effects of bullying. Additionally, it provides insights into the data collected during the COVID-19 pandemic concerning both forms of bullying. Accordingly, the study offers insights into the pandemic’s impact on youth risk behaviors attributable to the increased screen time among youths, which was a significant change during the pandemic. Hence, the findings of this study contribute to the existing body of evidence crucial for developing targeted interventions and prevention strategies to mitigate the harmful impacts of bullying in both physical and virtual settings. Moving forward, further investigation is warranted to delve into the nuanced interactions between sociodemographic and lifestyle factors and bullying victimization among populations beyond traditional school environments, to cultivate safer and more inclusive environments for all students. This is particularly critical because the recent uptick in AI use is expected to raise student’s use of digital devices and screen time.

## Figures and Tables

**Table 1:** Descriptions of independent variables. 2021 Youth Risk Behavior Surveillance System (YRBSS).

Variable	Survey Item	Response Choice
Dependent variables		
In-school bullied	During the past 12 months, have you ever been bullied on school property?	[no] or [yes]
Cyberbullied	During the past 12 months, have you ever been electronically bullied? (Count being bullied through texting, Instagram, Facebook, or other social media.)	[no] or [yes]
Independent variables		
Age group	How old are you?	14 years old or younger 15 years old 16 years old 17 years old 18 years old or older
Gender	What is your sex?	[female] or [male]
Race	The variable is computed from two questions: (1) Are Hispanic or Latino? and (2) What is your race?	White Black Asian Hispanic/Latino AI ^a^/AN ^b^ NH ^c^/other PI ^d^
Physical appearance of obesity	Had obesity (students who were ≥95th percentile for body mass index, based on sex- and age-specific reference data from the 2000 CDC ^e^ growth charts)	[no] or [yes]
Physical lifestyles of being physically active	Were physically active at least 60 min per day on 5 or more days (in any kind of physical activity that increased their heart rate and made them breathe hard some of the time during the 7 days before the survey)	[no] or [yes]
Physical lifestyles of spending a long time on digital games	Played video or computer games or used a computer 3 or more hours per day (counting time spent on things such as playing games, watching videos, texting, or using social media on your smartphone, computer, Xbox, PlayStation, iPad, or other tablet, for something that was not schoolwork, on an average school day)	[no] or [yes]
Risk-taken behaviors using marijuana/alcohol	Currently used marijuana	[no] or [yes]

^a^ AI = American Indian; ^b^ AN = Alaska Native; ^c^ NH = Native Hawaiian; ^d^ PI = Pacific Islander; ^e^ CDC = the Centers for Disease Control and Prevention.

**Table 2:** Distribution of U.S. high school students reporting being cyberbullied or in-school bullied by demographic and lifestyle factors, YRBSS, 2021 (Total = 17,232).

Variables		n ^a^ (Not Weighted)	% (Weighted)
Dependent variables			
Being cyberbullied (n = 17,032)	No	14,267	83.77
	Yes	2765	16.23
Being in-school bullied (n = 16,706)	No	13,994	83.77
	Yes	2712	16.23
Independent variables			
Age groups (n = 17,134)	≤14 years old	3504	20.45
	15 years old	4427	25.84
	16 years old	4276	24.96
	17 years old	3904	22.79
	≥18 years old	1023	5.97
Gender (n = 16,968)	Female	8152	48.04
	Male	8816	51.96
Race (n = 16,800)	White	9151	54.47
	Black	2322	13.82
	Asian	850	5.06
	Hispanic/Latino	1213	7.22
	AIAN ^b^	145	0.86
	NH/PI ^c^	88	0.52
	Multiracial	3031	18.04
Obesity appearance (n = 14,896)	No	12,341	82.85
	Yes	2555	17.15
At least 1 h of physical activity per day for 5 days during the past 7 days (n = 16,652)	No	7658	45.99
	Yes	8994	54.01
At least 3 h per day on video/computer games or computers (n = 16,496)	No	4064	24.64
	Yes	12,432	75.36
Currently using marijuana (n = 16,897)	No	14,250	84.33
	Yes	26.47	15.67

^a^ Numbers that do not add to the total (17,232) indicate missing values. ^b^ AIAN = American Indian or Alaska Native; ^c^ NH/PI = Native Hawaiian or other Pacific Islander.

**Table 3:** Chi-square analysis of factors associated with being cyberbullied or in-school bullied among high school students in the United States, YRBSS, 2021.

Variable	Category	Cyberbullied			In-School Bullied		
		No	Yes	*p* ^a^	No	Yes	*p* ^a^
Age group	≤14 years	2857	595	<0.001	2771	631	<0.001
	15 years	3600	776		3485	746	
	16 years	3556	681		3501	662	
	17 years	3313	556		3304	519	
	≥18 years	868	141		861	137	
Gender	Male	7723	975	<0.001	7415	1130	<0.001
	Female	6366	1720		6411	1507	
Race	White	7372	1700	<0.001	7236	1678	<0.001
	Black	2054	231		2047	207	
	Asian	729	114		736	88	
	Hispanic/Latino	1081	111		1060	110	
	AIAN ^b^	108	36		108	33	
	NH/PI ^c^	81	7		72	11	
	Multiracial	2505	495		2424	499	
Obesity appearance	No	10,307	1923	0.189	10,141	1851	0.001
	Yes	2109	425		2033	452	
More than an hour of physical activity per day for 5 days during the past 7 days	No	7352	1535	<0.001	7276	1481	0.006
	Yes	6447	1143		6270	1134	
More than 3 h a day on digital games or computers	No	3476	530	<0.001	3343	586	0.014
	Yes	10,206	2113		10,076	2003	
Current use of marijuana	No	1894	1992	<0.001	11,784	2060	<0.001
	Yes	1894	709		1955	596	

^a^ *p* = *p*-value; ^b^ AIAN = American Indian or Alaska Native; ^c^ NH/PI = Native Hawaiian or other Pacific Islander.

**Table 4:** Logistic regression analysis of victimization through cyberbullying or in-school bullying among high school students in the United States, YRBSS, 2021.

Variable	Category	Cyberbullying			In-School Bullying		
		AOR ^a^	*p* ^b^	95% CI ^c^	AOR ^a^	*p* ^b^	95% CI ^c^
Age group	≤14 years	Ref	Ref	Ref	Ref	Ref	Ref
	15 years	0.951	0.618	0.779–1.160	0.800	0.028	0.655–0.977
	16 years	0.829	0.074	0.675–1.018	0.703	0.001	0.574–0.861
	17 years	0.730	0.004	0.589–0.904	0.606	<0.001	0.491–0.749
	≥18 years	0.648	0.014	0.459–0.914	0.529	<0.001	0.372–0.753
Gender	Male	Ref	Ref	Ref	Ref	Ref	Ref
	Female	2.001	<0.001	1.735–2.322	1.380	<0.001	1.193–1.597
Race	White	Ref	Ref	Ref	Ref	Ref	Ref
	Black	0.344	<0.001	0.268–0.442	0.383	<0.001	0.296–0.495
	Asian	0.724	0.043	0.530–0.990	0.574	0.002	0.405–0.814
	Hispanic/Latino	0.432	<0.001	0.310–0.602	0.376	<0.001	0.268–0.530
	AIAN ^d^	1.013	0.966	0.550–1.867	0.787	0.455	0.421–1.474
	NH/PI ^e^	0.317	0.113	0.077–1.310	0.224	0.063	0.046–1.085
	Multiracial	0.662	<0.001	0.553–0.793	0.713	<0.001	0.594–0.855
Obesity appearance	No	Ref	Ref	Ref	Ref	Ref	Ref
	Yes	1.319	0.003	1.010–1.582	1.303	0.004	1.090–1.559
More than an hour of physical activity per day for 5 days during the past 7 days	No	Ref	Ref	Ref	Ref	Ref	Ref
	Yes	0.963	0.611	0.834–1.113	0.941	0.421	0.813–1.090
More than 3 h a day on digital games or computers	No	Ref	Ref	Ref	Ref	Ref	Ref
	Yes	1.247	0.014	1.046–1.487	1.056	0.527	0.891–1.252
Current use of marijuana	No	Ref	Ref	Ref	Ref	Ref	Ref
	Yes	2.150	<0.001	1.820–2.539	1.818	<0.001	1.529–2.162

Ref = reference category; ^a^ AOR = adjusted odds ratio; ^b^ *p* = *p*-value; ^c^ CI = confidence interval; ^d^ AIAN = American Indian or Alaska Native; ^e^ NH/PI = Native Hawaiian or other Pacific Islander.

## Data Availability

A publicly available dataset was analyzed in this study and can be found at https://www.cdc.gov/healthyyouth/data/yrbs/data.htm (accessed on 10 April 2024).
